# Genetic Variants in Matrix Metalloproteinases *MMP3* (rs3025058) and *MMP9* (rs3918242) Associated with Colonic Diverticulosis

**DOI:** 10.3390/medicina59112031

**Published:** 2023-11-17

**Authors:** Piotr Nehring, Grażyna Gromadzka, Miłosz Jastrzębski, Adam Przybyłkowski

**Affiliations:** 1 Department of Gastroenterology and Internal Medicine, Medical University of Warsaw, 02-097 Warsaw, Polandmilosz.jastrzebski@wum.edu.pl (M.J.); 2 Faculty of Medicine, Collegium Medicum, Cardinal Stefan Wyszyński University in Warsaw, Wóycickiego 1/3, 01-938 Warsaw, Poland; g.gromadzka@uksw.edu.pl

**Keywords:** colon, diverticulosis, genetic, metalloproteinase, polymorphism

## Abstract

*Background and Objectives*: Diverticulosis affects a significant portion of the elderly population, with age and lifestyle being established risk factors. Additionally, genetic predisposition is gaining recognition as a contributing factor. This pilot study sought to explore the frequency of genetic variants in matrix metalloproteinases (MMPs) 3, 9, and 12 in a population of colonic diverticulosis patients. *Materials and Methods*: The study encompassed 134 participants: 59 diagnosed with colon diverticulosis during colonoscopy and 75 healthy controls. The cases and controls were meticulously matched in terms of age and gender. We assessed the distribution of genetic variants *MMP3* rs3025058, *MMP9* rs3918242, and *MMP12* rs2276109 using the polymerase chain reaction–restriction fragments length polymorphism technique. *Results*: The *MMP9* rs3918242 allele T was notably more frequent in individuals with diverticulosis when compared with the control group (*p* < 0.03). Furthermore, it was associated with dominant (OR = 2.62; 95% CI: 1.24–5.56; *p* < 0.01) and co-dominant (OR = 2.10; 95% CI: 1.06–4.13; *p* < 0.03) genetic models. The *MMP3* rs3025058 5A/5A genotype was nearly twice as frequent in patients with diverticulosis, while the 6A/6A genotype was only half as common in this group. Conversely, no significant correlation was established between *MMP12* rs2276109 and colonic diverticulosis. *Conclusions*: Our study offers the first insight into a potential connection between genetic variants in MMPs and colon diverticulosis. Specifically, allele T of *MMP9* rs3918242 and allele 5A of *MMP3* rs3025058 appear to be linked to this condition. These findings indirectly suggest a role for extracellular matrix proteins in the pathogenesis of diverticulosis.

## 1. Introduction

Diverticulosis is defined as the presence of an asymptomatic diverticula in the colon. When symptomatic, the condition is diagnosed as a diverticular disease [1]. Colonic diverticulosis affects about 35–50% of the population in Western countries, with the prevalence increasing with age. The prevalence of diverticulosis is notably higher in the elderly. Colonic diverticulosis is diagnosed in up to 20% of 40–49 years old patients, escalating to 60% in patients aged 60, and reaching up to 75% in individuals over 80 years old [1].

Notably, several well-established risk factors are associated with diverticulosis, including low dietary fiber intake, obesity, and low physical activity [2,3,4]. In Western countries, particularly in North America and Europe, colonic diverticulosis is a prevalent health issue, with a significant proportion of the population affected. The Western diet, often high in processed foods, low in dietary fiber, and rich in red meat, has been associated with an increased risk of diverticulosis. In contrast, the epidemiology of colonic diverticulosis in Eastern countries, such as those in Asia, has traditionally shown lower prevalence. The Eastern diet typically includes fiber-rich food like vegetables, fruits, and whole grain products, which may contribute to a reduced risk of diverticulosis [2]. However, there is evidence that the prevalence of colonic diverticulosis is increasing in Eastern countries, likely due to changing dietary habits and lifestyle factors associated with urbanization and globalization.

There is substantial evidence pointing toward a genetic predisposition to diverticulosis [5]. Based on the findings from the previous studies, it has been estimated that genetic predisposition accounts for 40–53% of diverticular disease cases [6]. Recent studies have unearthed intriguing genetic insights into diverticulosis. Certain polymorphic variants in the tissue inhibitors of metalloproteinase gene (*TIMP*) *1* (rs4898) and 2 (rs8179090), as well as *COL3A1* (rs3134646), are more frequent in individuals with colonic diverticulosis [7,8]. Genome-wide association studies (GWAS) have pinpointed 53 potential genetic loci associated with an elevated risk of colon diverticulosis [9,10,11]. These studies collectively identified various genetic loci associated with diverticular disease, shedding light on potential pathomechanisms involving neuromuscular, connective tissue, and epithelial factors. The findings contribute to the growing body of knowledge regarding the genetic basis of diverticular disease, providing insights that may pave the way for improved diagnostics and targeted interventions. Interestingly, GWAS have not identified any associations with genes encoding laminins, matrix metalloproteinases (MMPs), or their inhibitors.

The increasing prevalence of colonic diverticulosis with age was shown to be intricately linked to the accumulation of collagen cross-links, which predispose the colon wall to the formation of weak spots [12,13]. Notably, the regulation of collagen and other proteins in the basal lamina is orchestrated by MMPs. Furthermore, diverticulosis is more frequently observed in patients with acquired and inherited connective tissue diseases, such as Marfan, Ehlers-Danlos, Coffin-Lowry, and Williams syndromes [5,14]. Connective tissue dysfunction contributes to the increased susceptibility of the colonic mucosa and submucosa to hernia formation at points of decreased resistance within the colon wall, particularly in areas surrounding the intramural *vasa recta*. This altered collagen composition significantly impacts the mechanical stability of connective tissue in individuals with diverticulosis and chronic inflammation [15]. Notably, higher expression of *MMP1* has been observed in patients with colonic diverticula [16]. In colonic diverticulosis, there is an increase in the synthesis of collagen type III and collagen cross-linking [12,17]. Additionally, collagen fibrils in the left colon become smaller and more densely packed with age, especially in colon diverticula, in contrast to those in the right colon, and this distinction is further accentuated [18].

The genotypes of *MMP3* −1612 5A/6A, *MMP9* −1561 C > T, and *MMP12* −82 A > G can potentially affect connective tissue through the expression and activity of MMPs. MMPs are enzymes that play a critical role in extracellular matrix (ECM) remodeling, including the degradation and synthesis of various components of connective tissue.

The *MMP3* gene encodes for matrix metalloproteinase 3, also known as stromelysin-1. The −1612 5A/6A polymorphism in this gene affects gene transcription and, subsequently, the production of MMP-3. The 5A allele is associated with higher transcription and production of MMP-3 compared with the 6A allele. The augmented expression of MMP-3 can lead to increased degradation of ECM components, such as proteoglycans and collagens, affecting the structural integrity of connective tissues, including cartilage and joint tissues. The 5A allele has been implicated in various conditions, including arthritis and degenerative joint diseases [19].

The *MMP9* gene encodes for matrix metalloproteinase 9, also known as gelatinase B. The −1561 C > T polymorphism affects gene expression and MMP-9 production. The T allele has been associated with higher MMP-9 expression compared with the C allele. Increased MMP-9 activity can contribute to tissue remodeling, potentially affecting the ECM in various tissues, including blood vessels and the extracellular matrix in the lungs. Elevated MMP-9 activity has been linked to conditions such as vascular diseases, pulmonary disorders, and tissue remodeling in response to injury or inflammation [20,21].

The *MMP12* gene encodes matrix metalloproteinase 12, also known as macrophage metalloelastase. The −82 A > G polymorphism may influence gene regulation and the production of MMP-12. Altered MMP-12 expression can affect connective tissue, as MMP-12 plays a role in elastin degradation and tissue remodeling. Changes in MMP-12 production due to genetic variations can impact the mechanical properties and elasticity of connective tissues, potentially contributing to diseases like emphysema and other lung-related conditions [22].

Given this intricate relationship between MMPs and colonic diverticulosis, this pilot study sought to investigate whether selected genetic variants in *MMP3*, *MMP9*, and *MMP12* are associated with the incidence of colonic diverticulosis.

## 2. Materials and Method

### 2.1. Patients

This study encompassed 134 patients who were admitted to the Department of Gastroenterology and Internal Medicine at the Medical University of Warsaw, Poland, spanning the period from 2017 to 2020. Patients were examined using a specially designed survey. Each patient underwent a comprehensive evaluation, including a colonoscopy, and their detailed medical history was meticulously documented. The diagnosis of diverticulosis, characterized by structural anomalies in the bowel involving submucosal herniations through weak areas of the smooth muscle without clinical symptoms, was established based on the findings from endoscopic examinations. These examinations adhered to the guidelines set forth by the World Gastroenterology Organization in 2007. Notably, only patients with left-sided diverticulosis were considered for inclusion, and individuals who had undergone colectomy or were diagnosed with symptomatic diverticular disease or diverticulitis were deliberately excluded from the study. All patients provided informed consent, and the study was ethically approved by the Ethics Committee of the Medical University of Warsaw, Poland.

### 2.2. Genetic Analysis

Whole blood samples were collected in EDTA tubes and stored at −80 °C. Genetic material was isolated from whole blood samples collected in EDTA tubes employing the Maxwell^®^ RSC Instrument and Maxwell^®^ 16 Blood DNA Purification Kit (Promega Corporation, Madison, WI, USA). The genotyping was carried out using the polymerase chain reaction-restricted fragments length polymorphism methodology (PCR-RFLP). Predesigned primers were utilized for the PCR process as follows: for *MMP3* rs3025058, forward 5′-GGTTCTCCATTCCTTTGATGGGGGGAAAGA-3′ and reverse 5′-CTTCCTGGAATTCACATCACTGCCACCACT-3′; for *MMP9* rs3918242, forward 5′-GCCTGGCACATAGTAGGCCC-3′ and reverse 5′-CTTCCTAGCCAGCCGGCATC-3′; and for *MMP12* rs2276109, forward 5′-GTCAAGGGATGATATCAGCT-3′ and reverse 5′-CTTCTAAACGGATCAATTCAG-3′ (Sigma-Aldrich, Inc., St. Louis, MO, USA). The PCR reactions were carried out using Color Taq PCR Master Mix (2×) (EURx Sp. z o.o., Gdańsk, Poland) and T100TM Thermal Cycler (Bio-Rad Laboratories, Inc., Philadelphia, PA, USA) following the manufacturer’s instructions. Subsequently, the PCR products were subjected to digestion with restriction enzymes: Tth111I (EURx Sp. z o.o., Gdańsk, Poland) for *MMP3* rs3025058, SphI (EURx Sp. z o.o., Gdańsk, Poland) for genotyping of *MMP9* rs3918242, and PvuII (EURx Sp. z o.o., Gdańsk, Poland) for *MMP12* rs2276109, in accordance with the previously described protocols [23,24,25].

### 2.3. Statistical Analysis

The statistical analysis was conducted using STATISTICA 13.3PL (TIBCO Software Inc., Palo Alto, CA, USA) software. To assess the normal distribution and variances in the data, the Kolmogorov-Smirnov and Shapiro-Wilk tests were employed. For variables exhibiting a normal distribution, means and standard deviations were presented and compared between groups using either one-way analysis of variance (for more than three groups) with post hoc analysis via the Neuman-Keulus test or the Student *t*-test (for two groups).

Variables that did not follow a normal distribution were presented as a median and interquartile range (IQR) and compared between groups using Kruskal-Wallis ANOVA (for more than three groups) with post hoc testing through the Mann-Whitney *U*-test with Bonferroni correction of *p* values or the Mann-Whitney *U*-test (for two-way variables). The effective genetic models were defined as dominant (RA + RR vs. AA), co-dominant (RR vs. RA vs. AA), and recessive (RR vs. AA + RA), with “R” representing the “risk allele”. The differences in allelic frequencies were assessed via a *χ*^2^ or Fisher’s exact test using PLINK software 1.9 (the Broad Institute, Cambridge, MA, USA) during the analysis of genetic associations to enhance the accuracy and reliability of the results. The Web-Assotest software (available at: http://www.ekstroem.com/assotest/assotest.html, accessed on 12 August 2022) and an online calculator from Institutüt für Humangenetik, Technische Universität München, Germany (available at: https://ihg.gsf.de/cgi-bin/hw/hwa2.pl, accessed on 12 August 2022) were also used for this purpose. To test for Hardy-Weinberg equilibrium (HWE) for studied polymorphisms, the expected genotype numbers were calculated from the allele frequencies, and deviation from the observed genotype numbers was determined using the *χ*^2^ test (the Hardy-Weinberg Calculator by Michael H. Court was used). Haplotype analysis was performed using Haplotype Analysis 1.05 software from Forest Genetics and Forest Tree Breeding at Georg-August-Universität Göttingen, Germany. Cases and controls were matched based on sex and age structure. Missing data were removed in pairs. The predetermined level of statistical significance was set at α = 0.05.

## 3. Results

Out of the 134 patients, 59 were diagnosed with diverticulosis, while the control group comprised 75 healthy individuals. The examination of the sex and age distribution within the comprehensive study population, encompassing both patients and controls, revealed a notable degree of comparability. The demographic breakdown exhibited a balanced representation, with the male-to-female ratios in the two groups being 21 to 38 and 26 to 49, respectively, and statistical analysis indicating no significant difference (*p* < 0.53). Similarly, the mean age of the cohorts demonstrated consistency, with values of 64.5 years ± 12.6 years for patients and 60.9 years ± 12.6 years for controls (*p* < 0.12), further underscoring the overall equilibrium in the composition of the studied populace. Examination of anthropometric parameters revealed no substantial distinctions between the cases and controls. The mean body mass was found to be 73.08 kg ± 17.36 kg for cases and 75.68 kg ± 15.86 kg for controls, with the between-group disparity proving statistically insignificant (*p* < 0.42). Similarly, mean height, exhibited no variance between the two groups, registering at 166.08 cm ± 10.23 cm for cases and 166.19 cm ± 12.49 cm for controls (*p* < 0.96). In terms of comorbidities, a comparison between cases and controls revealed no significant differences in the incidence of various medical conditions. The absence of statistically significant disparities encompassed a spectrum of health concerns, including abdominal hernias, diverticula in other parts of the gastrointestinal tract, hemorrhoidal disease, hypertension, ischemic heart disease, inflammatory bowel diseases, colon polyps, gastroesophageal reflux disease, thyroid gland diseases, and psoriasis.

Remarkably, none of the patients under investigation had been diagnosed with diverticulitis, and all instances of diverticulosis within the patient group were exclusively left-sided. Furthermore, the evaluation of patients using the Diverticular Inflammation and Complication Assessment score (DICA) yielded uniform results. All patients received a DICA score of 1, with a median score of 2 points and a range between 2 to 3 points.

The exploration of genetic predispositions through a comparison of risk allele frequencies (RAFs) in both cases and controls is intricately detailed in Table 1. Examining the frequency distribution of risk allele 5A of *MMP3* rs3025058, a distinctive pattern emerges, with cases exhibiting a frequency of 0.68, in contrast to controls where it stands at 0.59. Importantly, this observed difference maintains adherence to the Hardy-Weinberg equilibrium (HWE). The absence of available data on allele frequencies of *MMP3* rs3025058 in European population databases adds a layer of uniqueness to these findings. The frequency of the allele T of *MMP9* rs3918242 stands at 0.24, contrasting with controls where it is recorded at 0.11. This marked deviation from HWE (*p* < 0.03) prompts further exploration into the genetic dynamics at play. It is worth noting that the common RAF of allele T for *MMP9* rs3918242 in the broader European population is estimated at 0.17, highlighting the distinct genetic profile observed in the context of this study. The investigation into *MMP12* rs2276109 reveals a subtle contrast in risk allele frequencies. The frequency of risk allele A in cases is recorded at 0.84, closely mirroring the frequency in controls at 0.82. Importantly, this distribution aligns with the Hardy-Weinberg equilibrium, affirming the genetic stability in this regard. Notably, this observed frequency is consistent with the common estimated frequency of 0.88 in the broader European population.

**Figure 1:**
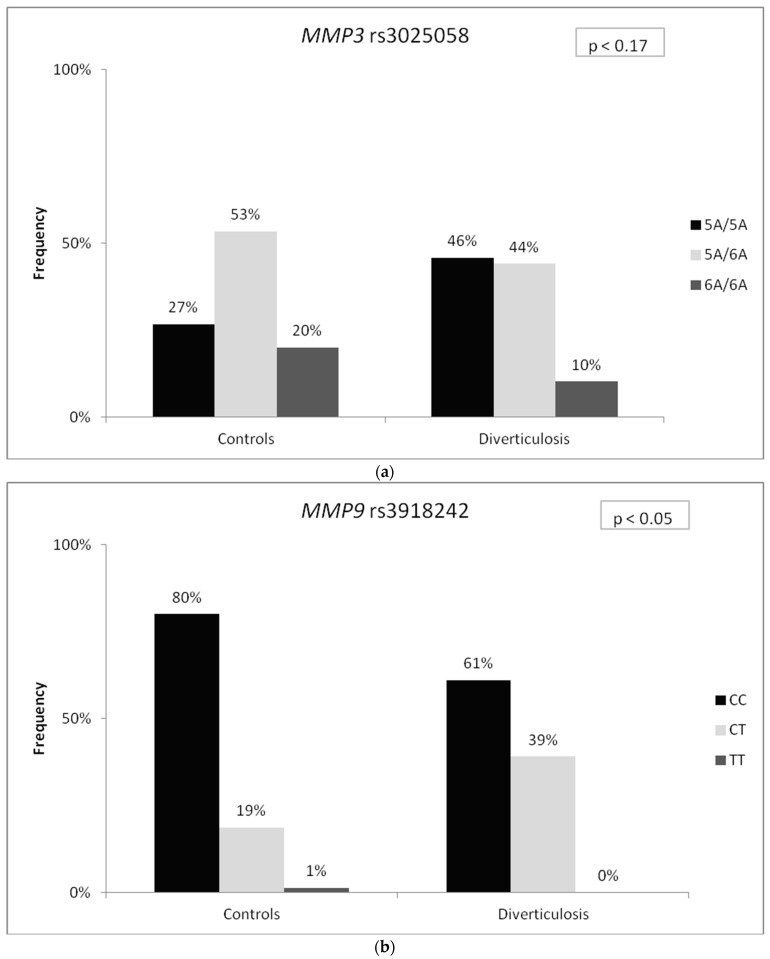

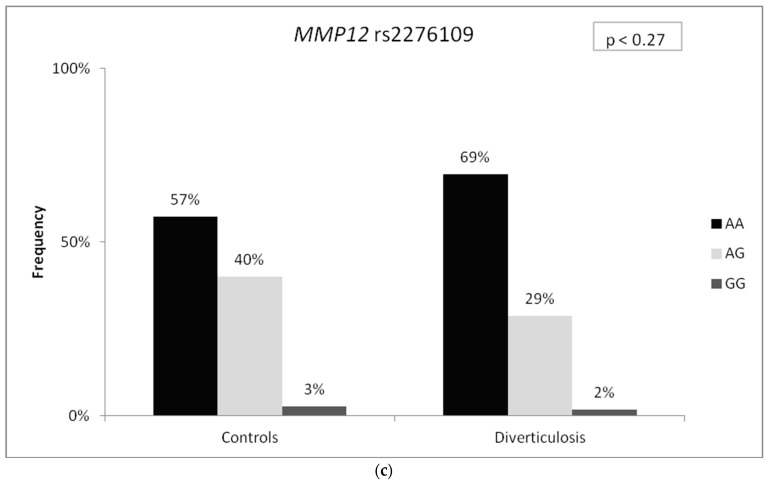
Analyzed genetic variants’ allele frequencies of *MMP3* rs3025058 (**a**), *MMP9* rs3918242 (**b**), and *MMP12* rs2276109 (**c**).

The frequency distribution of MMP risk alleles in cases and controls is detailed in Table 2. The frequency distribution of the *MMP3* rs3025058 5A/5A genotype was nearly twice as high and the 6A/6A genotype was halved among patients with diverticulosis compared with the control group, although this difference was of borderline statistical significance for the entire model. The genotype distribution of *MMP9* rs3918242 differed significantly between patients with diverticulosis and controls, with a higher frequency of the CT genotype and a lower frequency of the CC genotype (as illustrated in Figure 1a–c). Allele T of *MMP9* rs3918242 was more prevalent in patients with diverticulosis (*p* < 0.03) (Table 2). This allele showed significant associations in dominant and co-dominant genetic models, with odds ratios (OR) of 2.62 (95% CI: 1.24–5.56) and 2.10 (95% CI: 1.06–4.13), respectively (*p* < 0.01 and *p* < 0.03, respectively). The *MMP12* −82 A > G (rs2276109) genotype frequencies did not exhibit significant differences between patients and controls.

In the analysis of haplotypes involving three loci, the expected number of haplotypes was 27, but only 16 were observed (as shown in Table 3). Among patients with diverticulosis, the most prevalent haplotypes included *MMP3* −1715 5A/5A|*MMP9* −1562 C/C|*MMP12* −82 A/A (31%) and *MMP3* −1715 5A/6A|*MMP9* −1562 C/T|*MMP12* −82 A/G (14%). In contrast, controls exhibited a higher prevalence of haplotypes such as *MMP3* −1715 5A/6A|*MMP9* −1562 C/C|*MMP12* −82 A/A (24%) and *MMP3* −1715 5A/6A|*MMP9* −1562 C/C|*MMP12* −82 A/G (20%). Private haplotypes in individuals with diverticulosis included *MMP3* −1715 5A/5A|*MMP9* −1562 C/T|*MMP12* −82 G/G (2%) and *MMP3* −1715 6A/6A|*MMP9* −1562 C/T|*MMP12* −82 A/G (2%).

## 4. Discussion

In this study, we present compelling evidence that underscores the link between MMP genetic variants and diverticulosis. MMPs and their genetic variants may play a pivotal role in the pathogenesis of diverticulosis owing to their capacity to degrade extracellular matrix proteins [26,27].

We observed a increased prevalence of the *MMP3* −1715 5A allele surfaced among individuals with diverticulosis when juxtaposed with the control group. Notably, the 5A allele is intricately linked with elevated transcription rates and augmented production of MMP-3 in contrast to its counterpart, the 6A allele. Additionally, an increased frequency of the *MMP9* −1562 T allele manifested in patients with diverticulosis as opposed to their healthy counterparts. It is noteworthy that the T allele has been previously associated with heightened MMP-9 expression compared with its counterpart, the C allele. This collective elevation in MMP-3 and MMP-9 activity potentially engenders an escalated degradation of basal lamina proteins, thereby establishing a plausible predisposition to colonic diverticulosis. The intricate interplay of these genetic factors sheds light on the nuanced mechanisms underpinning the development of diverticulosis, offering valuable insights into potential avenues for therapeutic intervention and targeted management strategies.

Notably, haplotype analysis in patients with colonic diverticulosis unveiled the frequent coexistence of alleles, such as *MMP3* −1715 5A/5A (a genotype characterized by two alleles with high transcription activity), *MMP9* −1562 C/C (a genotype featuring two alleles with low transcription activity), and *MMP12* −82 A/A (a genotype with two alleles of high transcription activity). Strikingly, there was no coexistence of homozygous genotypes comprising two alleles with low transcription activity for *MMP12* and *MMP3* (i.e., *MMP12* −82 G/G and *MMP3* −1715 6A/6A), nor the coexistence of the low transcription activity *MMP12* −82 G/G genotype with the heterozygous genotype *MMP3* −1715 5A/6A (Table 3). This observation suggests that the presence of homozygous genotypes composed exclusively of alleles with either high or low transcriptional activity may be somehow deleterious.

MMP3, a stromelysin produced by fibroblasts and myofibroblasts, is proficient at breaking down basal lamina proteins, proteoglycans, E-cadherin, plasminogen, interleukin (IL)-1β, and chemokine 7 [28]. The *MMP3* rs3025058 is an upstream transcript variant that introduces an insertion (6A)/deletion (5A) variability at position −1612 within the promoter region. The *MMP3* rs3025058 6A promoter variant demonstrates reduced transcriptional activity due to its heightened repressor affinity for the binding site located in the polymorphic region [29]. In our study, diverticulosis patients exhibited a lower frequency of the *MMP3* rs3025058 6A/6A genotype in comparison with healthy controls. This reduced transcriptional activity of MMP3 may result in elevated degradation of basal lamina proteins related to MMP3 activity when compared with controls.

MMP9, known as neutrophil gelatinase, is produced by epithelium, fibroblasts, and leukocytes, and is capable of degrading elastin, type IV collagen, other basal lamina proteins, gelatins, tumor growth factor (TGF)-β, IL-8, and α1-antitrypsin [28]. The *MMP9* rs3918242 polymorphism represents a single nucleotide variation (SNV) resulting in a C to T substitution at position −1561 in the promoter region. *MMP9* rs3918242 has been associated with the risk of spontaneous deep intracerebral hemorrhage, potentially malignant and malignant lesions of the head and neck, and has served as a biological indicator of the efficacy of ulinastatin in the treatment of patients with severe acute pancreatitis [30,31,32]. The nucleoside C to T substitution in the promoter region of *MMP9* rs3918242 could potentially lead to a frameshift, thereby influencing the composition and function of the encoded protein, including alterations in proteolytic activity and gene expression. Both *MMP3* and *MMP9* are produced by fibroblasts and exhibit the capability to degrade basal lamina proteins. The genetic variants that promote higher activity of *MMP3* and *MMP9* or heightened production may contribute to increased degradation of basal lamina proteins and stimulate the formation of diverticula.

Given the relatively limited number of participants in this study, it can be characterized as a pilot study. As the first to establish an association between matrix metalloproteinase genetic variants and colonic diverticulosis, further investigations involving larger sample sizes are warranted to validate the findings presented here. The strength of this study lies in its homogeneous population from Central Europe, primarily characterized by diverticulosis limited to the left side of the colon. Subsequent research should consider encompassing patients with extended diverticulosis, diverticular disease, and diverticulitis to provide a comprehensive understanding of the genetic aspects of these conditions.

In further exploration of the genetic basis of diverticulosis, research should delve into the genetic variations in extracellular matrix (ECM) components such as collagens, elastin, fibronectin, laminins, proteoglycans, tenascins, and decorin. Additionally, the investigation should extend to the genetic variants in other MMPs, encompassing collagenases (MMP-1, MMP-8, and MMP-13), gelatinases (MMP-2 and MMP-9), stromelysins (MMP-3 and MMP-10), matrilysins (MMP-7 and MMP-26), and membrane-type MMPs (e.g., MT1-MMP/MMP-14), to comprehensively assess their potential roles in ECM degradation.

In addition to these findings, recent studies have explored the role of gut microbiota in diverticulosis pathogenesis, suggesting that dysbiosis may play an important role in diverticula formation [33]. Furthermore, emerging research has investigated the impact of dietary patterns, particularly those rich in prebiotic foods, on diverticulosis risk [34]. Numerous prebiotics fall under the category of dietary fiber, and a diet abundant in fiber is commonlyattributed with a low risk of diverticulosis. Sufficient fiber consumption contributes to the upkeep of regular bowel movements, serving as a preventive measure against constipation, a factor commonly associated with the formation of diverticula.

The study presented herein is a preliminary exploration underscoring the imperative to conduct further research using larger and more diverse cohort encompassing individuals afflicted by symptomatic diverticular disease and diverticulitis. Nevertheless, the presented results justify directing the search for diverticulosis genetic predisposition toward genes involved in degradation of connective tissue.

## 5. Conclusions

This study uncovers another link between genetic variants in matrix metalloproteinases and colonic diverticulosis. Patients with colonic diverticulosis exhibited a notably higher prevalence of Allele T in *MMP9* (rs3918242) and the 5A/5A genotype in *MMP3* (rs3025058) when compared with their healthy counterparts; the findings suggest a role for the degradation of ECM proteins in the formation of colonic diverticulosis. The presented results may open a new direction for further research in the pathology of diverticular disease.

## Figures and Tables

**Table 1:** Risk allele frequencies (RAFs) in the studied group.

	Risk Allele	RAF Cases	RAF Controls	HWE		dbGaP European
				*χ* ^2^	*p*	
*MMP3* rs3025058	5A	0.68	0.59	2.35	<0.13	N/A
*MMP9* rs3918242	T	0.24	0.11	4.89	<0.03	0.17
*MMP12* rs2276109	A	0.84	0.82	1.47	<0.22	0.88

dbGaP—National Center for Biotechnology Information database of Genotypes and Phenotypes for Allele Frequency Aggregator; HWE—Hardy-Weinberg equilibrium.

**Table 2:** Genetic associations of analyzed *MMP* variants for diverticulosis.

Genes (SNP ID)	Genotype, Allele	N (%) RAF *		corOR (95% CI)	*p* Value
		Diverticulosis (n = 59)	Controls (n = 75)		
*MMP3*	6A/6A	6 (10)	13 (17)	1.00	<0.41
−1612	5A/6A	26 (44)	36 (48)	1.57 (0.53–4.66)	
5A/6A	5A/5A	27 (46)	26 (35)	2.25 (0.74–6.81)	
(rs3025058)	5A (+)	0.68 *	0.58 *	1.48 (0.90–2.46)	<0.13
*MMP9*	C/C	36 (61)	61 (82)	1.00	<0.01
−1561	C/T	23 (39)	13 (17)	3.00 (1.35–6.64)	
C > T	T/T	0 (0)	1 (1)	---	
(rs3918242)	T (+)	0.24 *	0.11 *	2.18 (1.08–4.39)	<0.03
*MMP12*	G/G	1 (2)	2 (2)	1.00	<0.90
−82	G/A	17 (29)	29 (39)	1.17 (0.10–13.92)	
A > G	A/A	41 (69)	44 (59)	1.86 (0.16–21.34)	
(rs2276109)	A (+)	0.84 *	0.82 *	1.47 (0.79–2.75)	<0.22

* RAF, risk allele frequency.

**Table 3:** Haplotype frequencies in the studied groups.

Haplotype Code	Controls	Diverticulosis
*MMP3* −1715 5A/5A|*MMP9* −1562 C/C|*MMP12* −82 A/A	11%	31%
*MMP3* −1715 5A/5A|*MMP9* −1562 C/C|*MMP12* −82 A/G	5%	2%
*MMP3* −1715 5A/5A|*MMP9* −1562 C/C|*MMP12* −82 G/G	1%	0%
*MMP3* −1715 5A/5A|*MMP9* −1562 C/T|*MMP12* −82 A/A	4%	12%
*MMP3* −1715 5A/5A|*MMP9* −1562 C/T|*MMP12* −82 A/G	5%	0%
*MMP3* −1715 5A/5A|*MMP9* −1562 C/T|*MMP12* −82 G/G	0%	2%
*MMP3* −1715 5A/6A|*MMP9* −1562 C/C|*MMP12* −82 A/A	24%	12%
*MMP3* −1715 5A/6A|*MMP9* −1562 C/C|*MMP12* −82 A/G	20%	10%
*MMP3* −1715 5A/6A|*MMP9* −1562 C/T|*MMP12* −82 A/A	4%	8%
*MMP3* −1715 5A/6A|*MMP9* −1562 C/T|*MMP12* −82 A/G	4%	14%
*MMP3* −1715 5A/6A|*MMP9* −1562 T/T|*MMP12* −82 A/A	1%	0%
*MMP3* −1715 6A/6A|*MMP9* −1562 C/C|*MMP12* −82 A/A	12%	5%
*MMP3* −1715 6A/6A|*MMP9* −1562 C/C|*MMP12* −82 A/G	5%	2%
*MMP3* −1715 6A/6A|*MMP9* −1562 C/C|*MMP12* −82 G/G	1%	0%
*MMP3* −1715 6A/6A|*MMP9* −1562 C/T|*MMP12* −82 A/A	1%	2%
*MMP3* −1715 6A/6A|*MMP9* −1562 C/T|*MMP12* −82 A/G	0%	2%

## Data Availability

Dataset is available form the corresponding author on reasonable demand.
